# Defensive Compounds Involved in the Invasiveness of *Tithonia diversifolia*

**DOI:** 10.3390/molecules30091946

**Published:** 2025-04-27

**Authors:** Hisashi Kato-Noguchi, Midori Kato

**Affiliations:** Department of Applied Biological Science, Faculty of Agriculture, Kagawa University, Miki 761-0795, Kagawa, Japan

**Keywords:** allelochemical, herbivore, insect, invasive species, natural enemy, nematode, pathogen, sesquiterpene, tagitinin

## Abstract

*Tithonia diversifolia* (Hemsl.) A. Gray forms high-density impenetrable monospecific stands as an invasive plant species. Its life history traits, such as high adaptability with high genetic variation under different environmental conditions, and high growth and reproductive capacity, may contribute to its success in invading and increasing its population in the introduced ranges. Evidence has accumulated in the literature for the activity of compounds involved in the invasive characteristics of *Tithonia diversifolia* against natural enemies such as herbivorous insects and mammals, pathogenic nematodes, fungi, and viruses, and of allelochemicals against neighboring competitive plant species. Tagitinin A, tagitinin C, 1β-methoxydiversifolin, phytol, phytol acetate, α-pinene, bicyclo[3.1.0]hexane,4-methylene-1-(1-methylethyl), hispidulin, dihydro-*p*-coumaric acid, and methyl linoleate are toxic to herbivorous insects, and tagitinin C and 5-*O*-(*E*)-caffeoylquinic acid are harmful to herbivorous mammals. Tirotundin has nematocidal activity. α-Pinene, camphor, eucalyptol, and α-terpineol have fungicidal activity. Tagitinin A, tagitinin C, and 1β-methoxydiversifolin-3-*O*-methyl ether have antiviral activity. Tagitinin A, tagitinin C, 1β-methoxydiversifolin, and hispidulin act as allelochemicals that inhibit the growth of neighboring competing plant species. The ability to outcompete natural enemies and competing plant species is one of the essential factors for infestation and increase in the population and distribution of *Tithonia diversifolia* in new habitats. Therefore, these compounds may be involved in the invasive characteristics of *Tithonia diversifolia*. This is the first review article focusing on the compounds involved in the invasive characteristics of *Tithonia diversifolia.*

## 1. Introduction

**Figure 1:**
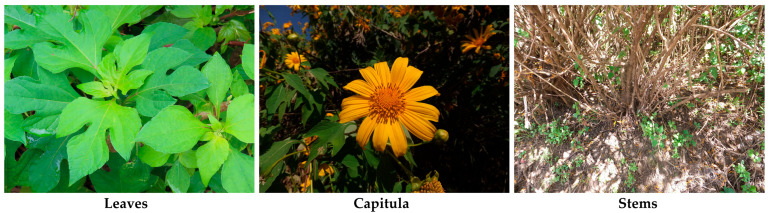
Leaves, capitula (flowers), and stems of *Tithonia diversifolia*.

*Tithonia diversifolia* (Hemsl.) A. Gray is a perennial shrub-like herbaceous plant in the Asteraceae family. It is known as the tree marigold and the Mexican sunflower. The species grows 2–4 m tall. The stems are green and hairy, becoming brown and woody with maturity, and are well branched. The leaves are alternate and subovate with 3–5 lobes, 10–40 cm long and 4–12 cm wide, and are attached to the stems by 5–15 cm long petioles. The capitula are 5–15 cm in diameter, consist of 7–15 ray florets and 80–120 disk florets, and are supported by pedicels (8–15 cm long) arising from the stems and branches. The corollas of the ray florets are yellow, elliptical, and 4–6 cm long. The fruits are dark brown flat achenes, 5–6 mm long [1,2,3,4] (Figure 1).

The native range of *Tithonia diversifolia* is Mexico and Central America, and it has been introduced as a horticultural plant and green manure, and as a contaminant of crop seeds [1,4,5,6,7]. *Tithonia diversifolia* populations have easily escaped to unintended locations, such as the crop fields, pastures, roadsides, open fields, riverbanks, forest edges, and protected areas including national parks [8]. The species has naturalized in more than 70 countries in the tropical, subtropical, and warm temperate regions of Australia, South America, Africa, Southeast Asia, and islands in the Pacific and Indian Oceans [1,2,3,4,7]. The first records of *Tithonia diversifolia* from eastern and southern Africa are herbarium specimens collected in Uganda in 1917 and in South Africa in 1927. Since then, *Tithonia diversifolia* has spread throughout eastern and southern Africa [8,9]. *Tithonia diversifolia* increased its population in South Africa by 40% between 2000 and 2016 [10]. *Tithonia diversifolia* was planted as green manure in Yunnan Province, China, and is thought to have escaped and naturalized in the 1930s [11,12]. Since then, populations have expanded, and the current distribution area in Yunnan Province is 72, 313–236, 673 km^2^ (difference due to estimation methods) at altitudes between 76 m and 2000 m, including 2822 km^2^ of protected areas [11,12,13]. *Tithonia diversifolia* has already naturalized in the coastal areas of northern New South Wales and Queensland, and on Christmas Island [3]. Under the current climatic conditions, *Tithonia diversifolia* may potentially spread to southern Europe and North America [14].

**Figure 2:**
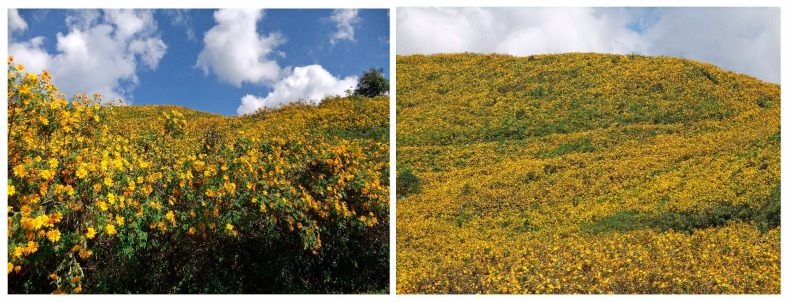
*Tithonia diversifolia* stands.

*Tithonia diversifolia* often forms high-density impenetrable monospecific stands. Plant density and aboveground biomass reach 8–20 plants per m^2^ and 22.4–31.6 kg fresh weight per m^2^, respectively [11,12]. The species outcompetes and displaces native vegetation [1,3]. *Tithonia diversifolia* infestation reduced species diversity from 59 to 44 species and the Shannon–Wiener diversity (a measure of the number of species and their abundance) by 27% in the lowland forest zone of Nigeria. The disappearance of several native plant species was observed in *Tithonia diversifolia*-infested plots. The Sorensen similarity index (an indication of the overlap between two populations) between infested and uninfested plots was 32.6% [15]. Infestation of *Tithonia diversifolia* was also observed to reduce species richness in Goromonzi, Zimbabwe [16], and Aizawl, North India [17]. Infestation of *Tithonia diversifolia* reduced water availability and crop production in agricultural fields and increased labor costs for managing the species [5,8]. It has been reported that many farmers in Nigeria have abandoned farmland infested with *Tithonia diversifolia* because of the difficulty of its management [18]. Due to its invasiveness, *Tithonia diversifolia* has been classified as a successful invader of new habitats [1,2,3,4] (Figure 2).

*Tithonia diversifolia* (chromosome 2n = 34) shows high genetic variation [19,20] and high adaptability to different environmental conditions, such as annual precipitation ranging from 1000 mm to 2000 mm, mean annual temperature ranging from 15 °C to 31 °C, and sandy soils to clay soils with pH values between 6.1 and 7.8. The species tolerates average annual temperatures from 12 °C to 38 °C, and annual precipitation from 700 mm to 2500 mm [1,14].

*Tithonia diversifolia* reaches reproductive maturity 4 months after germination, and flowers throughout the year under suitable conditions. *Tithonia diversifolia* grown under experimental field conditions had 35–212 capitula per plant with 32–62 seeds per capitulum, producing 1120–13,144 seeds per plant with an average of 897,342 seeds per square meter [21]. *Tithonia diversifolia* in the naturalized population had 755 capitula per plant with 180 seeds per capitulum, giving 135,900 seeds per plant [22]. The mean dry weight of 100 seeds was 0.5–0.7 g [6,21,22]. Under competitive conditions with neighboring plants, *Tithonia diversifolia* allocated more nutrients to reproductive activities than to vegetative activities and produced more seeds [6].

The seeds are light and can be effectively dispersed by wind, water, animals, or as contaminants in grain and soil [6,22]. The germination rate of *Tithonia diversifolia* seeds immediately after collection from the field was 16.3% but increased to 97.5% after storage for 4 months [22], suggesting after-ripening dormancy [23]. Soil samples were collected at a depth of 0–15 cm from roadside plots where *Tithonia diversifolia* had previously dominated fields, and the plant species emerging from the soil were monitored under greenhouse conditions. During the 6-month observation period, 87–193 *Tithonia diversifolia* seedlings emerged (1533–3401 seedlings/m^2^), indicating that *Tithonia diversifolia* had established an efficient seed bank for the next generation. *Tithonia diversifolia* also increases its population through vegetative production. When the stems and branches lie on the soil surface, nodal roots emerge from the nodes of the stems and branches. When the rooted nodes detach from the mother plants, physiologically independent clonal plants are formed [12]. Vegetative reproduction promotes the horizontal expansion and formation of dense monospecific stands. Therefore, *Tithonia diversifolia* has high reproductive capacity through prolific seed production and vegetative expansion, and high adaptive capacity to different environmental conditions with high genetic variation. These characteristics of the species may contribute to the naturalization and population increase of *Tithonia diversifolia* in new habitats.

*Tithonia diversifolia* has been reported to exhibit anti-herbivore activity against insects, snails, and mammals, anti-pathogen activity against nematodes, fungi, bacteria, and viruses, and allelopathic activity, and to contain compounds involved in these activities. Allelopathic activity increases the ability to compete with neighboring plant species for resources. Herbivores, pathogens, and neighboring competing plant species are universal biotic stressors for any plant species, and the defense functions against these biotic stressors have been suggested to be one of the essential functions for the successful naturalization and expansion of invasive plants in their introduced range [24,25,26,27,28,29,30,31]. However, no previous review article has focused on the defense functions and related compounds specific to *Tithonia diversifolia*. This is the first review article to provide an overview of the anti-herbivore and anti-pathogen activity of *Tithonia diversifolia*, including allelopathic activity, and the compounds involved in these activities. The literature was searched using a combination of major online search engines: Scopus, ScienceDirect, and Google Scholar, including all possible combinations of *Tithonia diversifolia* with the following words: botany, distribution, ecology, invasion, adaptation, habitat, impact, growth, reproduction, seed, clonal, herbivore, vertebrate, invertebrate, nematocidal, insecticidal, fungicidal, pathogen, allelopathy, allelochemical, and pharmacology.

## 2. Protection Against Herbivorous Insects

Herbivory is one of the most important detrimental biotic factors affecting plant fitness under natural conditions. Leaf loss of 10% due to herbivory does not seem to be serious. However, experimental defoliation of 10% caused significant reductions in growth, flowering, seed production, and produced seed viability in *Piper arietinum* [32,33]. Annual survival was 85% for undamaged seedlings of *Dipteryx panamensis*, and 0% for seedlings with only 8% defoliation [34]. Herbivorous insects also exert significant selective pressure on plant abundance and distribution, reducing plant growth, biomass, and seed production, and increasing leaf senescence and plant mortality [35,36,37]. Therefore, plants need to employ morphological and/or chemical defense strategies against the feeding activity of herbivorous insects [38,39,40,41]. In addition to morphological defense strategies, many invasive plant species have been reported to have evolved chemical defense strategies and to produce certain compounds with insecticidal activity [42,43]. Herbivorous insects are divided into specialists and generalists based on their feeding preferences. The specialists feed on a single plant species or a small number of plant species, and the generalists feed on a wide variety of plant species [35,36]. Specialist insects that feed only on the invasive plant species are likely to be few in the introduced ranges of invasive plants, due to the lack of co-evolutionary history between the invasive plants and the insects [44,45]. However, some of the generalist insects may feed on invasive plant species in their introduced ranges [35,36].

Aqueous, ethanol, and dichloromethane extracts of the *Tithonia diversifolia* leaves increased the mortality of a generalist herbivore, the leafcutter ant *Atta cephalotes* workers [46,47]. The dichloromethane extracts inhibited the activity of acetylcholinesterase in vitro with 50% inhibition at an extract concentration of 73.9 μg/mL [47]. Acetylcholine is an essential neurotransmitter in the central nervous system, and acetylcholinesterase catalyzes the conversion of acetylcholine to choline and acetate [48,49]. Inhibition of acetylcholinesterase leads to the accumulation of acetylcholine at the synapses, and keeps the acetylcholine receptor open, resulting in increased nerve excitation, and leading to the death of the organism [49,50,51]. These ants cut plant leaves and cultivate the symbiotic fungus *Leucoagaricus gongylophorus* on the cut leaves in their nests for food [52,53]. When *Tithonia diversifolia* leaves were given to *Atta cephalotes* for its leaf-cutting activity, the growth of the symbiotic fungus was inhibited, and the mortality of *Atta cephalotes* increased [54,55].

Aqueous extracts of the *Tithonia diversifolia* leaves suppressed the population of the aphid *Aphis gossypii* on *Solanum tuberosum* (potato) plants. This aphid is widely distributed with a very wide host range and suck the sap from the host plants [56]. Aqueous extracts of the leaves of *Tithonia diversifolia* showed repellent activity against the adult whitefly *Aleurodicus dugesii* and increased mortality with LD_50_ 3.1 mg/L [57], and increased the mortality of the sap-sucking psyllid *Diaphorina citri* [58]. Methanol extracts of the leaves of *Tithonia diversifolia* increased the mortality of the moth larvae *Crocidolomia pavonana* [59], and adult termites of *Ancistrotermes* spp. with LD_50_ 29.76 mg/L [60]. Essential oil from the leaves of *Tithonia diversifolia* increased the mortality of the aphid *Aphis gossypii*, onion thrips *Thrips tabaci*, and silverleaf whitefly *Bemisia tabaci*. These insects are widely distributed, and have a wide host range, including agricultural crops. *Thrips tabaci* and *Bemisia tabaci* also transmit plant diseases by feeding [61].

The larvae of the sunflower patch *Chlosyne lacinia*, whose host plants belong to the Heliantheae tribe, including *Tithonia diversifolia*, feed only on the part of the *Tithonia diversifolia* leaves where the density of glandular trichomes on the abaxial side is very low and avoid feeding on the remaining parts of the leaves. Leaf rinse extracts also showed antifeedant activity against the larvae. The extracts of the glandular trichomes and leaves contained several sesquiterpene lactones, and tagitinin C was the major constituent of both extracts [62]. These results suggest that the glandular trichomes contain tagitinin C, and that tagitinin C may be responsible for the antifeedant activity.

The painted lady *Vanessa cardui* is the most widely distributed butterfly, and its larvae feed on a variety of plants, including members of the Asteraceae family. When the larvae fed on the leaves of *Tithonia diversifolia*, their development was retarded and their mortality increased. Although several phenylpropanoids, flavones, and sesquiterpene lactones were identified in the leaves of *Tithonia diversifolia*, tagitinin C may be responsible for the feeding deterrent effect against *Vanessa cardui* [63]. *Tithonia diversifolia* leaves were soaked in dichloromethane for 3 min, and the soaked solution was applied onto the leaves of various plant species. After the solvent dried, the larvae of the cotton bollworm *Helicoverpa armigera*, which feeds on a wide range of plants including many crops, were placed on the leaves and their feeding activity was monitored. The soaked solution showed antifeedant activity and increased larval mortality. The main component in the extracts was tagitinin C, which may have been responsible for the activity of the soaked solution [64].

**Figure 3:**
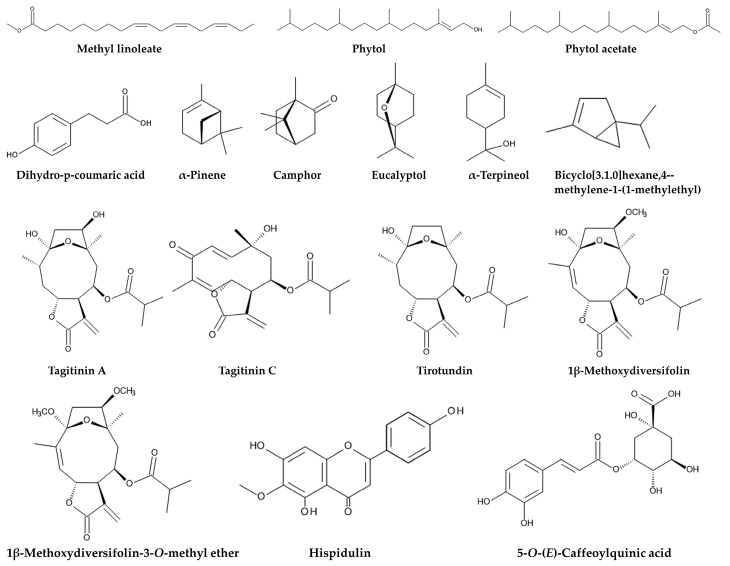
Compounds involved in the defense functions against herbivorous insects and mammals, parasitic nematodes, pathogenic fungi and viruses, and competing plant species.

Tagitinin A and C and hispidulin isolated from chloroform extracts of the aerial parts of *Tithonia diversifolia* showed antifeedant activity against moth larvae of the eri silkworm *Samia ricini* [65]. Ethyl acetate extracts of the leaves of *Tithonia diversifolia* suppressed the development of the larvae of the fall armyworm *Spodoptera frugiperda* and increased larval mortality. The main active components in the extracts were tagitinin A, tagitinin C, and 1β-methoxydiversifolin. These compounds showed this activity at the concentrations higher than 10 ppm [66]. Moth larvae *Spodoptera frugiperda* feed on a variety of plant species including crops such as wheat, soybean, corn, soybean, and cotton [67]. The two-spotted spider mite *Tetranychus urticae* is widely distributed and infests over 1200 plant species including crops, fruits, and ornamentals [68,69]. Ethyl acetate and methanol extracts of *Tithonia diversifolia* leaves increased the mortality of *Tetranychus urticae* and inhibited its oviposition. The ethyl acetate extracts showed higher oviposition activity. Tagitinin A and tagitinin C were the major components in both extracts, and the ethyl acetate extracts contained more tagitinin A and tagitinin C [70] (Figure 3).

In addition, essential oil of the leaves of *Tithonia diversifolia* increased the mortality of stored grain pests including the rice weevil *Sitophilus oryzae* and the flour beetle *Tribolium castaneum*. α-Pinene (63%) and bicyclo[3.1.0]hexane,4-methylene-1-(1-methylethyl) (14.8%) were the major components of the essential oil [71]. Dihydro-*p*-coumaric acid isolated from the leaves of *Tithonia diversifolia* increased the mortality of *Sitophilus oryzae*, *Rhyzopertha dominica*, and *Tribolium castaneum.* The LD_50_ value after 74 h of application of dihydro-*p*-coumaric acid was 11.49 μg/L, 10.29 μg/L, and 17.80 μg/L for *Sitophilus oryzae*, *Rhyzopertha dominica*, and *Tribolium castaneum*, respectively. Dihydro-*p*-coumaric acid also showed inhibitory activity on the acetylcholinesterase of these insects, resulting in the disruption of the synapse transmitting system [72]. Dichloromethane and ethyl acetate extracts of the *Tithonia diversifolia* leaves increased the mortality of the corn weevil *Sitophilus zeamais*. The efficacy of the dichloromethane extract was greater than that of the ethyl acetate extract. The major components in both extracts were methyl linoleate, phytol, and phytol acetate. The concentration of phytol acetate in the dichloromethane extracts was 2.9 times higher than that in the ethyl acetate extracts [73]. Methanol extracts of *Tithonia diversifolia* leaves increased the mortality of the cowpea beetle *Callosobruchus maculatus.* Tagitinin A was isolated from the active fractions of the extracts and showed toxicity to *Callosobruchus maculatus* [74]. According to the literature, *Tithonia diversifolia* has anti-insect activity and contains several compounds involved in this activity. Anti-insect activity is one of the essential factors for invasive plants to successfully naturalize into their introduced range and increase their population [25,26,75,76,77,78,79].

## 3. Protection Against Herbivorous Snails

Methanol extracts of *Tithonia diversifolia* leaves showed molluscicidal activity against the generalist herbivorous snail *Pomacea canaliculata* [80]. This snail is listed in the top 100 of the World’s Worst Invasive Alien species [81]. *Pomacea canaliculata* feeds on a wide variety of plant species and causes significant problems in agricultural production [82]. Therefore, *Tithonia diversifolia* may contain certain compounds with molluscicidal activity. However, the compounds involved in this activity have not yet been reported. In addition, no information is available on the relationship of *Tithonia diversifolia* with herbivorous invertebrates other than herbivorous insects and snails.

## 4. Protection Against Herbivorous Mammals

Herbivorous mammals can cause damage to plant growth and survival by consuming plants [37,38]. Oral administration of the aqueous extracts of *Tithonia diversifolia* leaves to the Wistar rat *Rattus norvegicus* (200–250 g body weight) at a dosage of 100 mg/kg for 14 days altered hematological parameters such as total proteins, albumin, creatinine, triglycerides, erythrocytes, alkaline phosphatase, and aspartate transaminase activity. Histological analysis showed severe steatosis in the liver and lesions in the kidneys of these rats. Tagitinin C and 5-*O*-(*E*)-caffeoylquinic acid were identified as toxic substances in the extracts [83]. Oral administration of aqueous ethanol extracts of the aerial parts of *Tithonia diversifolia* also showed toxic effects on the liver and kidneys of *Rattus norvegicus* (average weight 170 g). Significant periportal hepatocyte damage was observed after 30 min of administration. The LD_50_ value was 1600 mg/kg per day [84]. The aqueous extracts of *Tithonia diversifolia* were toxic to the albino rat *Rattus norvegicus albinus*. After 14 days of oral administration at a dosage of 200–800 mg/kg, the extracts caused significant increases in the levels of serum alanine aminotransferase, alkaline phosphatase, and aspartate aminotransferase. Histological analysis showed that the extracts caused severe damage to the liver and kidneys of the rats [85]. Dietary supplementation (15–20%) of *Tithonia diversifolia* leaves significantly increased the activity of serum glutamic pyruvate transaminase and serum glutamic oxaloacetate transaminase in the grasscutter *Thryonomys swinderianus* (a larger rodent, 830–850 g body weight). However, this supplemental diet had no apparent adverse effects on the growth and health status of the grasscutters [86] (Figure 3).

*Tithonia diversifolia* leaves were fed as a supplemental diet to Holstein x Zebu dairy cows (519 kg body weight) for 21 days. The inclusion (15.4%) of *Tithonia diversifolia* leaves in a dairy cow diet had no effect on milk production and quality, nor on the serum concentrations of glucose, urea nitrogen, and cholesterol [87]. Although up to 20% inclusion of *Tithonia diversifolia* leaves in a dairy diet for hair sheep *Ovis aries* showed no toxic effect, higher levels of inclusion (35% and 50%) in the diets increased nitrogen loss in urine and feces [88]. *Tithonia diversifolia* is used as livestock feed in several tropical regions due to its high mineral and nutrient values [89,90]. However, toxicity of *Tithonia diversifolia* to livestock such as goats, cattle, and sheep, including death, has been observed by the local farmers [8]. It may be important to consider the ratio of *Tithonia diversifolia* inclusion in dairy diets for livestock to avoid its toxic effects.

Thus, *Tithonia diversifolia* may contain substances that are toxic against herbivorous mammals. These toxic substances cause liver and kidney damage and are more effective in the relatively small mammals. These compounds may protect *Tithonia diversifolia* from herbivory by small mammals.

## 5. Protection Against Parasitic Nematodes

Many of the parasitic nematodes feed on the plant roots, and the feeding process causes significant damage to the plant root system, reducing the ability to absorb nutrients and water and the ability to defend against infection by other pathogens [91,92,93]. The root-knot nematodes *Meloidogyne* spp. are widely distributed and have a wide range of host plant species. Juveniles of the root-knot nematodes invade plant root cells, form a permanent feeding site consisting of multiple giant cells, and induce gall formation in the host plant roots. Through these giant cells, the nematodes extract nutrients and water from the host plants and cause severe disease, including wilting, yellowing, and/or stunting. Among the parasitic nematodes, root-knot nematodes have the most significant effects on plant growth and regeneration [94,95]. The reniform nematodes *Rotylenchulus* spp. also form a permanent feeding site and cause stunting, necrosis, and/or chlorosis on a wide range of the host plant species. The root lesion nematodes *Pratylenchus* spp. and the burrowing nematodes *Radopholus* spp. cause necrosis by migration into plant cells and feeding. Root lesion nematodes and burrowing nematodes do not form a permanent feeding site and can move from parasitized roots into the soil and infest other roots [96,97]. Therefore, the ability of invasive plants to defend themselves against parasitic nematodes may be critical to their success in naturalizing and spreading in new habitats.

Incorporation of leaf, stem, and root powder of *Tithonia diversifolia* into the soil reduced the population of *Meloidogyne incognita* and its gall formation in the roots of *Solanum lycopersicum* (tomato) [98] and in the roots of *Vigna unguiculata* [99]. Compost of *Tithonia diversifolia* suppressed the infection by the parasitic nematode *Pratylenchus brachyurus* in *Zea mays* (maize) [100]. Methanol extracts of the leaves of *Tithonia diversifolia* reduced the hatching and movement of *Meloidogyne incognita* juveniles and their gall formation in the roots of *Canephora canephora* (coffee) [101]. Aqueous extracts of the *Tithonia diversifolia* leaves also reduced egg hatching, population, and gall formation of *Meloidogyne incognita* [102] and the development of this nematode and its parasitism on *Amaranthus cruentus* and *Solanum macrocarpon* under greenhouse conditions [103]. Aqueous extracts of the leaves of *Tithonia diversifolia* also suppressed the proliferation and development of the reniform nematode *Rotylenchulus* spp. [104]. In addition, four-week-old *Solanum lycopersicum* plants were inoculated with *Meloidogyne incognita*, dipped in the aqueous extracts of the *Tithonia diversifolia* leaves for five minutes, and grown for 60 days under greenhouse conditions. The treatments resulted in suppression of the population and gall formation of *Meloidogyne incognita* in the roots of *Solanum lycopersicum* [105]. Thus, *Tithonia diversifolia* may contain certain compounds with anti-nematode activity that are extractable with methanol and water.

The intercropping of *Tithonia diversifolia* plants in *Phaseolus vulgaris* (common bean) fields reduced the population and parasitism of *Meloidogyne incognita* and *Meloidogyne javanica* in *Phaseolus vulgaris* roots. After three months of growing *Tithonia diversifolia* in the experimental fields, the *Tithonia diversifolia* plants were removed from the fields. *Phaseolus vulgaris* was then planted in the same fields, which resulted in a reduction in the population and parasitism of *Meloidogyne incognita* and *Meloidogyne javanica* in *Phaseolus vulgaris* roots [106]. The intercropping of *Tithonia diversifolia* in a *Musa* spp. (banana) plantation reduced the parasitism of the burrowing nematode *Radopholus simili* and the root-lesion nematode *Pratylenchus coffeae* in the roots of *Musa* spp. [107]. Therefore, *Tithonia diversifolia* plants may release nematocidal compounds into the field soil and these compounds can reduce the population and parasitism of these nematodes.

Tirotundin isolated from the aerial parts of *Tithonia diversifolia* inhibited the acetylcholinesterase activity of the free-living nematode *Caenorhabditis elegans*, with an LD_50_ value of 9.16 μg/mL [108]. Inhibition of acetylcholinesterase disrupts synapse transmission in the nervous system [49,50,51]. Pesticides such as carbamates and organophosphates also inhibit acetylcholinesterase and are classified as acetylcholinesterase inhibitors [109] (Figure 3). Thus, *Tithonia diversifolia* may contain some nematocidal agents, and tirotundin may be one of them.

## 6. Protection Against Fungal, Bacterial and Viral Pathogens

Disease is also one of the most important deleterious biotic factors affecting plant fitness. Pathogenic fungi, bacteria, and viruses exert significant selective pressure on plant abundance and distribution, increase senescence and reduce plant growth, biomass, reproduction, and survivorship [38,77,110,111]. Therefore, defense functions against such pathogens are essential for invasive plant species to expand their distribution and increase their population in new habitats. Many invasive plant species have been reported to have evolved chemical defense strategies against pathogen infection, and to produce certain compounds that have anti-pathogen activity [112,113].

Aqueous extracts of *Tithonia diversifolia* leaves significantly suppressed the growth of the pathogenic fungus *Colletotrichum gloeosporioides* at an extraction rate of 120 g of leaves per one liter of water. The inhibitory activity of the extracts at this concentration was equivalent to the commercial fungicide Antracol at 2 g/L, which is the recommended concentration for the fungicide application [114]. Aqueous extracts of the leaves of *Tithonia diversifolia* suppressed infection with *Colletotrichum capsici*, which causes leaf blight [115,116]. Aqueous extracts of the leaves of *Tithonia diversifolia* suppressed the infection with *Mycosphaerella fijiensis*, which causes leaf-spot known as black Sigatoka disease on *Musa* spp. [117]. Ethanol, methanol, and acetone extracts of *Tithonia diversifolia* leaves suppressed the necrotrophic pathogen *Alternariabrassicicola*, which causes black spot disease on a wide range of host plant species. The inhibitory activity of the ethanol extract was the greatest among the three extracts [118]. Aqueous extracts of *Tithonia diversifolia* leaves suppressed the growth of *Bipolaris oryzae*, which causes brown spots [119], and *Fusarium oxysporum* and *Fusarium moniliforme* [119,120]. *Fusarium* spp. are widely distributed in soil and cause wilt, blight, canker, rot, and root necrosis. *Fusarium* spp. are known as necrotrophic fungi that kill tissue to extract nutrients from plants [121]. Necrotrophic pathogens cause necrosis and even death of the infected plants [122,123]. Essential oil obtained from the leaves of *Tithonia diversifolia* also suppressed the growth of *Bipolaris oryzae*, *Fusarium moniliforme*, and *Xanthomonas oryzae*, which cause leaf blight, and *Pseudomonas fuscovaginae*, which causes brown sheath rot. The major constituents of the essential oil were α-terpineol (20.3%), eucalyptol (cineole, 14.6%), camphor (14.3%), and α-pinene (13.5%) [119].

Ethanol extracts of the leaves and flowers of *Tithonia diversifolia* inhibited infection by the pathogenic bacterium *Ralstonia solanacearum*, which causes wilt disease in a wide range of host plants [124]. Tagitinin C and 1β-methoxydiversifolin-3-*O*-methyl ether were isolated from the methanol extracts of whole plants of *Tithonia diversifolia* as anti-tobacco mosaic virus (TMV) agents. Tagitinin C and 1β-methoxydiversifolin-3-*O*-methyl ether suppressed the infection of *Nicotiana tabacum* (tobacco) plants by TMV. Tagitinin C and 1β-methoxydiversifolin-3-*O*-methyl ether inhibited the TMV gene expression of the coat protein and RNA-dependent RNA polymerase in tobacco cells. Both gene expressions are essential for TMV infection into the host plant cells [125]. The pathogenic virus *Orthotospovirus* (TAWV) causes wilting and spotting diseases in more than 1000 monocotyledonous and dicotyledonous plant species [126,127]. Tagitinin A suppressed the TAWV infection in *Nicotiana tabacum*. Tagitinin A inhibited TAWV gene expression of in tobacco cells. Tagitinin A also increased the jasmonic acid level and the gene expression of *NtCOI1* (*Nicotiana* coronatine-insensitive protein 1) [128]. Jasmonic acid is a plant hormone involved in the defense function against pathogen infection, and *NtCOI1* is the marker gene of the jasmonic acid signaling pathway [129,130]. In this context, tagitinin A induces the defense function by activating the jasmonic acid signaling pathway, which suppresses the gene expression of TSWV. Tagitinin A also induced the gene expression of F-box protein (CPR30; component of ubiquitin protein ligase E3), and activated the defense function against the pathogen infection [128] (Figure 3).

Based on the literature, *Tithonia diversifolia* has antifungal, antibacterial, and antiviral activity and contains several compounds involved in these activities. α-Terpineol, eucalyptol, camphor, and α-pinene may protect *Tithonia diversifolia* from infection by pathogenic fungi, and tagitinin A, tagitinin C and 1β-methoxydiversifolin-3-*O*-methyl ether may protect *Tithonia diversifolia* from infection by pathogenic viruses. Tagitinin A induces the defense function by activating the jasmonic acid signaling pathway. Therefore, these compounds may contribute to the invasive characteristics of *Tithonia diversifolia.*

## 7. Protection Against Competition from Neighboring Plant Species

Plants fight with neighboring plants for the acquisition of resources such as light, water, nutrients, and niches. Plants that have strong competitive ability against neighboring plants win a relatively large amount of resources. Therefore, the competitive ability of the plants is one of the important factors for the increase in their population and distribution [25,27,131,132]. Many invasive plants have been reported to have relatively high allelopathic activity and to contain several allelochemicals [27,28,131,132,133]. Allelochemicals are biosynthesized, accumulated in plant tissues and released into the environment as needed through secretion, volatilization, and the degradation processes of plant tissues in the soil [134,135,136,137]. The released allelochemicals affect the neighboring competing plant species, suppressing their emergence, growth, and fitness. As a result, these plant species acquire stronger competitive ability and gain an advantage in resource competition with the neighboring plant species [138,139,140,141,142]. Allelochemicals of the invasive plants have been identified in extracts of plant parts, in root exudates, essential oils, decomposing plant residues, and rhizosphere soils [29,43,143,144].

When aqueous extracts (10%, *w*/*v*) of the *Tithonia diversifolia* leaves were sprayed on the field soil (10 L/ha) of a *Vigna unguiculata* (cowpea) cropping system, the total weed density in the field decreased by 63.7% compared with the control treatments, and the grain yield of cowpea increased by 71.2% [145]. Aqueous extracts of *Tithonia diversifolia* shoots suppressed the growth of *Bidens pilosa* [146], the germination and growth of *Tridax procumbens* [147], growth, including leaf and chlorophyll development, in tree plant species *Hildegardia barteri*, *Dialium guineense*, and *Monodora tenuifolia* under field conditions [148], and germination and growth parameters such as plant weight, leaf area, plant height, and root length of *Amaranthus cruentus* under greenhouse and field conditions [149]. Aqueous methanol extracts of *Tithonia diversifolia* leaves also suppressed the seedling growth of *Lolium multiflorum*, *Echinochloa crus-galli*, and *Phleum pratense* [150] and the growth of *Sorghum bicolor* [151]. Thus, *Tithonia diversifolia* may be allelopathic and contain certain allelochemicals. These allelochemicals are extractable with water and methanol.

The root exudates of *Tithonia diversifolia* inhibited the germination, growth, and leaf development of *Amaranthus dubius* under greenhouse conditions [152]. Field soil from *Tithonia diversifolia* stands suppressed the emergence of the weed species *Panicum maximum*, *Bidens pilosa*, *Acanthospermum hispidum*, *Pennisetum polystachyon*, and *Euphorbia heterophylla* under greenhouse conditions [153] and the seedling growth of *Cyperus iria*, *Digitaria ciliaris*, and *Amaranthus viridis* [154]. In addition, aqueous extracts of the soil from *Tithonia diversifolia* stands inhibited the seedling growth of *Cyperus iria*, *Digitaria ciliaris*, and *Amaranthus viridis* [155]. Therefore, *Tithonia diversifolia* may release certain allelochemicals as root exudates into the soil under *Tithonia diversifolia* stands. Some allelochemicals may also accumulate in the soil during the decomposition of *Tithonia diversifolia* residues.

An allelochemical was isolated from aqueous methanol extracts of *Tithonia diversifolia* leaves using a bioassay-guided purification procedure. During the bioassay-guided purification process, the biological activity of all fractions obtained from each separation step was evaluated, and the most active fraction was subjected to the next separation step. The chemical structure of the isolated allelochemical was tagitinin C. Tagitinin C suppressed the growth of *Lolium multiflorum*, *Echinochloa crus-galli*, and *Phleum pratense* at concentrations greater than 0.1 mM, and the concentrations of tagitinin C required for 50% growth inhibition were 0.35–0.83 mM [150]. Tagitinin A and hispidulin were also isolated from the aerial parts of *Tithonia diversifolia* [156]. Fourteen compounds were isolated from the ethyl acetate extracts of whole plants of *Tithonia diversifolia*, and tagitinin A, tagitinin C, and 1β-methoxydiversifolin were the major constituents of the extracts [157] (Figure 3).

Based on the literature, *Tithonia diversifolia* is allelopathic and synthesizes and releases several allelochemicals into the environment, including the rhizosphere. The release of allelochemicals results in the suppression of the emergence, growth, and/or fitness of the neighboring competing plant species. Based on the novel weapon hypothesis, allelochemicals are more effective against neighboring plants in the introduced range than in the native range of the invasive plant species. The competing plant species in the native range may have evolved the ability to cope with the suppressive effect of the allelochemicals during the long periods of the co-evolutional history between them. However, the plant species in the introduced ranges may not have such a co-evolutional history and may not have acquired the tolerance to the allelochemicals [131,132]. Therefore, the allelochemicals in *Tithonia diversifolia* may be more effective in the plant’s introduced ranges. However, some of the compounds have been evaluated only under laboratory conditions. The activity of the compounds should be evaluated under field conditions to assess whether these compounds contribute as allelochemicals to the expansion of the population of *Tithonia diversifolia* in new habitats.

## 8. Compounds Involved in the Invasive Properties of *Tithonia diversifolia*

*Tithonia diversifolia* produces several defense compounds against herbivorous insects and mammals. Tagitinin A, tagitinin C, 1β-methoxydiversifolin, and hispidulin increased insect larval mortality and suppressed larval feeding and development and adult oviposition [62,63,64,65,66,70]. α-Pinene, bicyclo[3.1.0]hexane,4-methylene-1-(1-methylethyl), phytol, phytol acetate, dihydro-*p*-coumaric acid, and methyl linoleate increased the mortality of stored grain pests [71]. Dihydro-*p*-coumaric acid showed inhibitory activity of acetylcholinesterase [72]. Tagitinin C and 5-*O*-(*E*)-caffeoylquinic acid increased the serum levels of alanine aminotransferase, alkaline phosphatase, aspartate aminotransferase, glutamic pyruvate transaminase, and/or glutamic oxaloacetate, and caused liver and kidney damage in rats and rodents [83] (Table 1).

*Tithonia diversifolia* produces several defense compounds against pathogens such as nematodes, fungi, bacteria, and viruses. Tirotundin inhibited the acetylcholinesterase activity of nematodes [108] and disrupted the synapse transmitting system. α-terpineol, eucalyptol, camphor, and α-pinene suppressed the population growth of pathogenic fungi [119]. Tagitinin C and 1β-methoxydiversifolin-3-*O*-methyl ether inhibited the TMV gene expression of the coat protein and RNA-dependent RNA polymerase and suppressed TMV infection [125]. Tagitinin A increased F-box protein gene expression and jasmonic acid levels [128]. F-box protein and jasmonic acid are involved in the defense function against the pathogen infection. *Tithonia diversifolia* also produces allelochemicals against neighboring competitive plant species. Tagitinin A, tagitinin C, 1β-methoxydiversifolin, and hispidulin inhibited the growth of several other plant species [150,156,157].

Phytochemical investigations suggest that *Tithonia diversifolia* contains many other compounds, such as monoterpenes, sesquiterpenes, flavonoids, and anthraquinones [158,159,160,161,162]. Some of these compounds have shown pharmacological activities, such as anti-inflammatory effects, analgesic effects, antidiabetic effects, antiprotozoal effects, antibacterial effects, antifungal effects, and antiviral effects [160,161,162,163,164,165]. Although the contributions of these compounds to the plant’s defense functions have not yet been evaluated, it is possible that some of these compounds may be involved in the invasive characteristics of *Tithonia diversifolia*, with unknown functions. The concentration of sesquiterpene lactones in *Tithonia diversifolia* increases with an increase in temperature [166,167]. Therefore, the global warming trend may activate the production of sesquiterpene lactones, such as tagitinin A, tagitinin C, tirotundin, 1β-methoxydiversifolin, and 1β-methoxydiversifolin-3-*O*-methyl ether, and enhance the defense functions of *Tithonia diversifolia*, which may result in the increased infestation of *Tithonia diversifolia* in new habitats. Some of these compounds may synergistically contribute to the plant’s defense functions and increase the invasiveness of *Tithonia diversifolia*, although these data are not available in the literature.

## 9. Conclusions

*Tithonia diversifolia* is naturalized in more than 70 countries from tropical to warm temperate regions. The species forms high-density impenetrable monospecific stands that reduce species abundance and diversity in the infested areas and affects agricultural crop production. The life history traits of *Tithonia diversifolia*, such as high adaptability to different environmental conditions with high genetic variation, and its high growth and reproductive capacity, may contribute to increases in population size and distribution in new habitats. *Tithonia diversifolia* produces several compounds involved in defense functions against its natural enemies, such as herbivorous insects and mammals, pathogenic nematodes, fungi, and viruses, and competing plant species. The ability to defend against natural enemies and competing plant species is one of the essential factors for invasion and survival. Therefore, these compounds may be involved in the invasive characteristics of *Tithonia diversifolia* and contribute to the success of the species in naturalizing and increasing its population and distribution in new habitats. 

However, the mechanical actions of not all of the compounds have been determined, and some other compounds may also be involved in the invasiveness of *Tithonia diversifolia.* Their mechanical actions and these compounds should be investigated in the future.

## Figures and Tables

**Table 1:** Compounds and functions of *Tithonia diversifolia*.

Phytochemical Class		Defense Function Against					Reference
	Compound	Insect	Mammal	Nematode	Fungus, Virus	Competing Plant	
Fatty acid	Methyl linoleate	✓					[73]
Hydroxycinnamic acid	Dihydro-*p*-coumaric acid	✓					[72]
Monoterpene	α-Pinene	✓			✓		[71,119]
	Bicyclo[3.1.0]hexane,4-methylene-1-(1-methylethyl)	✓					[71]
	Camphor				✓		[119]
	Eucalyptol				✓		[119]
	α-Terpineol				✓	✓	[119]
Sesquiterpene	Tagitinin A	✓			✓	✓	[65,66,70,74,128,150,156,157]
	Tagitinin C	✓	✓		✓	✓	[62,63,64,65,66,70,83,125,150,157]
	Tirotundin			✓			[108]
	1β-Methoxydiversifolin	✓					[66,157]
	1β-Methoxydiversifolin-3-*O*-methyl ether				✓		[125]
Diterpene	Phytol	✓					[73]
	Phytol acetate	✓					[73]
Flavonoid	Hispidulin	✓				✓	[65,156]
Polyphenol	5-*O*-(*E*)-Caffeoylquinic acid		✓				[83]

## Data Availability

Not applicable.
